# Is there a non-linear relationship between dietary protein intake and prostate-specific antigen: proof from the national health and nutrition examination survey (2003–2010)

**DOI:** 10.1186/s12944-020-01234-6

**Published:** 2020-05-02

**Authors:** Jukun Song, Chi Chen, Song He, Weiming Chen, Jiaming Su, Dongbo Yuan, Fa Sun, Jianguo Zhu

**Affiliations:** 1 Department of Oral and Maxillofacial Surgery, Guizhou Provincial People’s Hospital, Guiyang, 550001 Guizhou China; 2 Medical College of Guizhou University, Guiyang, 550001 Guizhou China; 3 Department of Immunology and Microbiology, Guiyang College of Traditional Chinese Medicine, Guiyang, 550001 Guizhou China; 4 Department of Urology, Guizhou Provincial People’s Hospital, The Affiliated Hospital of Guizhou Medical University, Guiyang, 550001 Guizhou China

**Keywords:** Dietary protein intake, Prostate-specific antigen (PSA), NHANES database

## Abstract

**Background:**

Growing evidence demonstrated that dietary protein intake may be a risk factor for prostate cancer and elevate the level of prostate-specific antigen (PSA). However, proof for the correlation between dietary protein intake and PSA in American adults without prostate tumor history is limited. Our goal was to investigate the association of dietary protein intake with PSA using the National Health and Nutrition Examination Survey (NHANES) (2003–2010) database.

**Methods:**

After the screening, 6403 participants were included in the study. The interested independent is the dietary protein intake, and the dependent variable is PSA levels, the covariates included demographic, dietary, biological data, and physical examination variables. A weighted linear model and a weighted linear regression model were used to examine the distribution of variables in the covariate differences between the different independent groups according to quartiles. Four models were used to survey the association between dietary protein intake and PSA. We also attempted to find a nonlinear relationship between dietary protein intake and PSA using the GAM model and the penalty spline method and further solved the nonlinear problem using weighted two-piecewise linear model.

**Results:**

The weighted multivariate linear regression analysis demonstrated that dietary protein intake was not independently associated with PSA levels after adjusting potential confounders (β = 0.015, 95%CI:-0.024, 0.055). However, we found the non-linear relationship between dietary protein intake and PSA, whose point was 18.18 g (per 10 g change). The magnitude and confidence intervals for the left and right inflection points are − 0.03 (− 0.09, 0.02) and 0.22 (0.07, 0.36), respectively. On the right side of the inflection point, one gram of increment in protein intake was associated with increased PSA levels by 0.22 (log2 transformation: 0.22, 95%CI: 0.07, 0.36).

**Conclusions:**

After adjusting for potential covariates, the non-linear correlation between dietary protein intake and PSA was observed. When dietary protein intake exceeded the threshold of 181.8 g, dietary protein intake was positively correlated with elevated PSA levels.

## Introduction

Prostate cancer (PCa) remains the most commonly diagnosed cancer in men in the world. PCa is regarded as the second most common cause of cancer-related death for men in the world [1]. In 2018, approximately 164,690 new cases and 29,430 deaths were estimated to be associated with PCa in the United States [2]. The incidence rate of prostate cancer is higher in Western countries and it’s have risen sharply, together with mortality, in the past decades [3, 4]. A large number of studies strongly suggest that environmental factors play a key role in the pathogenesis of PCa. It is speculated that the prevalence of PCa in Western countries is largely due to the fundamental dietary characteristics of Western diet patterns [5, 6], which are characterized by high intake of protein and fat, as well as refined carbohydrates. Growing evidence indicated that dietary protein restriction (PR) diet is associated with lower PCa incidence [7, 8].

Due to the increasing incidence of PCa worldwide, strengthening early screening and diagnosis of PCa can help reduce mortality [3, 4]. The screening of PCa population is mainly based on Prostate-specific antigen (PSA). Therefore, the factors affecting PSA must be clarified to ensure the quality of screening and avoid missed diagnosis. PSA is a useful tumor marker for PCa and has been widely used as a screening tool for the disease [9, 10]. PSA is essentially a serine protease and has been widely used in clinical practice as a screening tool for PCa since 1988. It is the most well-known member of the Kalli-Kerin family with a 24% positive predictive value as a screening tool for the detection of PCa [11, 12]. Known recognized risk factors such as age, prostatitis, certain drugs such as 5-alpha reductase inhibitors (5ARIs) and prostate size can affect PSA levels [13]. In recent studies, dietary protein restriction may affect PSA levels [14, 15]. Understanding how PSA is associated with specific mechanisms that contribute to cancer, such as changing dietary model, can improve future screening methods. To date, there is still a lack of evidence regarding the association of PSA and dietary protein intake in the general population. Therefore, we performed a secondary data analysis based on existing data that comes from the public NHANES data. We aim to explore the relationship between dietary protein intake and PSA level. In addition, we assessed whether an increase or change in protein intake would affect PSA levels.

## Methods

### Data source

Since 1960, the National Centers for Disease Control and Prevention (CDC) National Center for Health Statistics has conducted a National Health and Nutrition Examination Survey (NHANES) every two years to provide national estimates of the health and nutritional status of non-institutional populations in the United States. Data from the official website of NHANES (https://wwwn.cdc.gov/nchs/nhanes/Default.aspx) is available for free download. The NHANES protocol was reviewed and approved by the National Center for Health Statistics research ethics review board. All participants received written informed consent. More detailed information about NHANES can be found on the official website.

### Study population

**Fig. 1:**
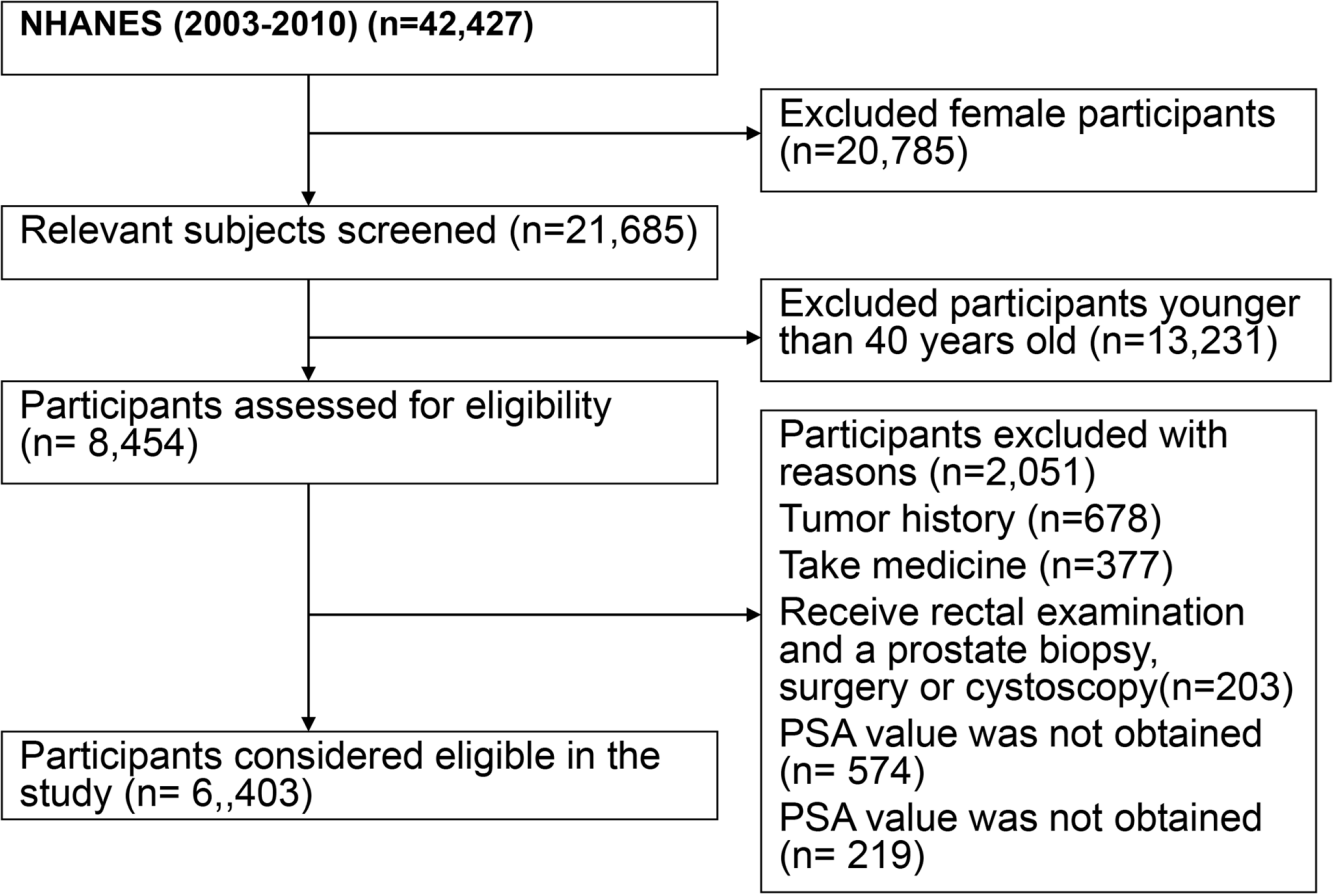
Flow chart of procedures from identification of eligible patients to final inclusion

The NHANES database only has PSA data for 2003–2010, therefore we integrated data from four two-year NHANES survey cycles: 2003–2004, 2005–2006, 2007–2008 and 2009–2010, and performed secondary data analysis. We restricted the population included in the analysis to men 40 years of age and older and did not have a history of prostate tumor [16]. They provided blood samples for PSA assessment as part of NHANES. The participants were screened according to the following exclusion criteria: (1) Men with prostate cancer, prostatitis, or recent prostate surgery (ie, a rectal exam within 1 week, and a prostate biopsy within 1 month, surgery or cystoscopy) were not included in the study. (2) We also excluded men who used 5ARI or other forms of hormone therapy (ie, testosterone replacement or medical castration) and drugs, with incomplete clinical or socio-demographic data. After a series of screening, 6403 out of 42,470 participants were included in the study. The detailed flowchart is shown in Fig. 1.

### Variables

In the current study, the targeted independent variable was dietary protein intake (gm). The US Department of Agriculture (USDA) Automatic Multiple Pass Method (AMPM) was used to collect dietary intake data by interviewers 24 h a day. A detailed description of the dietary interview method has been described elsewhere [17]. The targeted dependent variable was PSA (ng/mL). For the present study, serum PSA concentration (ng/mL) was measured using the Beckman Access Immunoassay System with the Hybritech Total PSA Assay (Beckman Coulter, Fullerton, CA) [18].

Covariates were selected based on previous studies demonstrating the link between these covariates and dietary protein intake and/or prostate cancer/PSA [16, 19]. Covariates included demographic, dietary, biological, and immunological variables. Variables included in the database file were as follows: continuous variables included LDL-cholesterol (mg/dL), Poverty income ratio (PIR), Body mass index (Kg/m2), Total alcohol intake on the first day (gm), Vitamin D (ng/mL), C-reactive protein(mg/dL), Glycohemoglobin (%), HDL-cholesterol (mg/dL), cigarettes per day during past month, Age (year), Total protein intake on the first day (gm) and Triglycerides (mg/dL). Categorical variables consisted of race, hypertension history, diabetes history, coronary heart disease, stroke, education level, marital status, physical activity, and enlarged prostate. In general, covariates relate to demographic data, dietary data, physical examination data, and comorbidities in the NHANES database. A more detailed explanation of the variables can be found on the NHANES official website.

### Statistical analysis and missing data

We conducted a statistical analysis according to the criteria of the CDC guidelines (https://wwwn.cdc.gov/nchs/nhanes/tutorials/default.aspx). In order to enhance the statistical strength, we transformed the dietary protein intake by per 10 g change as the targeted independent variable, and we use log2 transformation and use the transformed data as the independent variable for data analysis because PSA is skewed distribution. Continuous variables were expressed as mean ± standard deviation (normal distribution) or median (quartile) (skewed distribution), and categorical variables were expressed in frequency or as a percentage. To investigate whether dietary protein intake is related to PSA levels in selected participants, our statistical analysis consists of three main steps. Firstly, the dietary protein intake was divided into four groups according to the quartile levels and presented the distribution of baseline data of patients included in this study in different dietary protein intake groups (Quartile). The chi-square tests (categorical variables), One –Way ANOVA (normal distribution), or Kruskal-Wallis test (skewed distribution) was used to demonstrate for differences among four quartile groups. In the second step of data analysis, the weighted univariate and multivariate linear regression model was employed. Four statistical models were constructed: model I, no covariates were adjusted; model II, only adjusted for socio-demographic data; model III, model 2 + other covariates exhibited in Table 1, model IV, a weighted generalized additive model (GAM). The third step of data analysis was to conduct the GAM model and smooth curve fitting (penalized spline method) to explore the nonlinearity association between dietary protein intake and PSA levels. If the GBM model detects nonlinearity, we first calculate the inflection point using a recursive algorithm and then construct a weighted two-stage linear regression model on both sides of the inflection point. We determined the best fit model based on the *P*-value of the log-likelihood ratio test (linear regression model and two piecewise linear regression models).

**Table 1:** Baseline characteristics of selected participants

Dietary Protein (gm)	Q1(2.94- 58.89)	Q2 (58.91- 82.25)	Q3( 82.29-111.50)	Q4 (111.51-399.74)	*P* value
Prostate-specific antigen (PSA,ng/ml) log2 transform	0.13 (-3.84-5.32)	0.04 (-3.84-5.32)	-0.14 (-3.84-5.32)	-0.18 (-3.32-5.32)	<0.0001
**Sociodemographic variables**					
Age,year	64.00 (40.00-85.00)	61.00 (40.00-85.00)	57.00 (40.00-85.00)	51.00 (40.00-85.00)	<0.0001
Poverty income ratio	2.35 (1.53)	2.68 (1.60)	2.93 (1.61)	3.02 (1.66)	<0.0001
**Race/Ethnicity (%)**					<0.0001
Mexican American	7.12	7.03	6.08	6.58	
Other Hispanic	3.77	2.66	4.11	2.83	
Non-Hispanic White	68.35	75.38	77.75	78.60	
Non-Hispanic Black	14.05	10.39	8.57	8.47	
Other Race - Including Multi-Racial	6.70	4.53	3.50	3.53	
**Education level (%)**					<0.0001
less than high school	13.74	8.92	5.91	4.89	
high school	42.14	34.95	36.01	34.88	
more than high school	44.12	56.13	58.08	60.23	
**Marital status (%)**					<0.0001
Married	65.93	70.55	74.46	72.33	
single	30.22	24.99	20.74	21.01	
Living with partner	3.85	4.45	4.80	6.66	
**Variables of laboratory data**					
Body mass index, mean ± SD (Kg/m2)	28.44 (5.52)	28.45 (5.99)	29.08 (5.52)	29.38 (5.71)	<0.0001
Alcohol first day, mean ± SD (gm)	9.09 (25.55)	12.01 (27.90)	15.58 (35.78)	20.48 (41.62)	<0.0001
C-reactive protein, mean ± SD (mg/dL)	0.21 (0.01-18.50)	0.20 (0.01-13.70)	0.19 (0.01-13.90)	0.18 (0.01-10.50)	<0.0001
Glycohemoglobin (%)	5.98 (1.22)	5.93 (1.22)	5.88 (1.18)	5.79 (1.05)	<0.0001
HDL-C, mean ± SD (mg/dL)	46.00 (19.00-145.00)	46.00 (20.00-148.00)	45.00 (15.00-144.00)	46.00 (19.00-179.00)	0.5658
Smoked at least 100 cigarettes in life					0.0013
Yes	62.00	59.06	58.02	54.99	
No	38.00	40.94	41.98	45.01	
Triglycerides (mg/dL)	160.77 (125.46)	174.83 (148.83)	181.55 (169.46)	179.40 (148.52)	0.0023
Hypertension history					0.0029
Yes	42.44	41.20	36.86	35.26	
No	57.56	58.80	63.14	64.74	
Diabetes history (%)					<0.0001
Yes	18.19	12.24	11.08	9.67	
No	81.81	87.76	88.92	90.33	
coronary heart disease (%)					<0.0001
Yes	10.19	8.03	4.55	4.04	
No	89.81	91.97	95.45	95.96	
Stroke (%)					<0.0001
Yes	5.88	4.01	3.22	1.52	
No	94.12	95.99	96.78	98.48	
Physical activity (%)					0.0002
Sits	26.00	25.86	23.31	20.71	
Walks	50.95	49.43	48.24	44.26	
Light loads	14.46	17.43	19.20	22.87	
Heavy work	8.59	7.28	9.24	12.16	
Enlarged prostate (%)					0.0586
Yes	15.92	16.66	15.75	12.72	
No	84.08	83.34	84.25	87.28	

Missing data addressing is needed for the accuracy of data analysis because a series of variables in the NHANES database have different degrees of missing. If only using complete case for data analysis, it will cause a large number of samples to be lost and may produce bias in our findings. Therefore, we have adopted multiple interpolations, the main purpose of which is to maximize statistical power and minimize bias that might occur covariates with missing data were excluded from data analyses. We created 5 imputed datasets with chained equations using a mice software package. In addition, we used sensitivity analysis to identify whether created complete data had a significant difference from pre-imputation data. Our findings demonstrated that created complete data showed no significant difference from raw data. Therefore, all results of our multivariable analyses were based on the imputed datasets and were combined with Rubin’s rules.

To ensure the robustness of data analysis, we did the following sensitivity analysis: (1) we converted the dietary protein intake into a categorical variable by quartile and calculated the P for trend. The purpose was to verify the results of dietary protein intake as a continuous variable and to observe the possibility of nonlinearity; (2) we employed the weighted GAM model to adjust the continuous variables in model III.

All analysis was performed using statistical software R (http://www.r-project.org, The R Foundation) and EmpowerStats (http://www.empower-stats.com, X&Y Solutions, Inc., Boston, MA). A *p*-value of less than 0.05 (two-sided) was considered statistically significant.

## Results

### Baseline characteristics of participants

Baseline characteristics of selected participants from NHANES 2003 to 2010 according to quartiles of dietary protein intake are exhibited in Table 1. There was no statistically significant difference the distribution of HDL, cigarettes per day during the past month and Enlarged prostate in four dietary protein intake groups (quartiles, Q1-Q4) (all *p* values > 0.0 5).

Compared to Q4 group, subjects with high dietary protein intake were older, had lower Vitamin D intake, LDH, Poverty income ratio, Body mass index, Alcohol first day, Protein first day and Triglycerides. In contrast, participants in other groups(Q1-Q3) has higher C-reactive protein and Glycohemoglobin levels, Physical activity, reported a higher incidence of hypertension, Diabetes, coronary heart disease, stroke. Most of the participants were Non-Hispanic White population.

### Dietary protein intake and PSA levels

The magnitude of the correlation between Dietary protein intake and PSA levels was listed in Table 2. We used the imputation data to summarize the effect sizes of the Model 2, 3 and GAM models through Rubin rules (see Supplementary Tables 1 and 2 for details). Model 1 is an unadjusted model. Model 1 indicated that for each additional unit of dietary protein intake, the PSA level is reduced by 0.028 (0.036–0.021) with P for trend less than 0.05. In Model 2, after adjusting for socio-demographic variables (Race/Ethnicity, Poverty income ratio, Age, year, marital status, education level), the association between dietary protein intake and PSA level was not significant with P for trend > 0.05. In fully-adjusted mode, after adjusting for Vitamin D intake (mcg), LDL-cholesterol (mg/dL), Race/Ethnicity, Poverty income ratio, Body mass index (Kg/m2), Alcohol (gm) first day, C-reactive protein (mg/dL); Glycohemoglobin (%), HDL, Hypertension history, Diabetes history, coronary heart disease, stroke, cigarettes per day during past month, Age (year), Marital Status, Average level of physical activity each day, Enlarged prostate, Triglycerides (mg/dL), education level, marital status, the association between dietary protein intake and PSA level was still not significant with P for trend > 0.05. To solve the nonlinear problem, we also use GAM to adjust the continuous variables in the covariate. Despite these transformations (fitting continuous variables to smoothing), the results did not change significantly (model 4).

**Table 2:** Univariate and multivariate analysis by weighted linear regression model and GAM model

Exposure	Non-adjusted model	Minimally-adjusted model	Fully-adjusted model	GAM model
PSA (ng/ml) log2 transform				
Dietary Protein (gm)	-0.028 (-0.036, -0.021) <0.00001	-0.002 (-0.010, 0.005) 0.52921	0.015 (-0.024, 0.055) 0.44749	0.023 (-0.018, 0.064) 0.28372
Dietary Protein (quartile)				
Q1	0	0	0	
Q2	0.002 (-0.101, 0.105) 0.96758	0.098 (-0.003, 0.200) 0.05821	0.238 (-0.392, 0.868) 0.46152	0.383 (-0.244, 1.009) 0.23490
Q3	-0.194 (-0.293, -0.094) 0.00015	0.006 (-0.095, 0.106) 0.91227	-0.552 (-1.105, 0.001) 0.05422	-0.315 (-0.914, 0.284) 0.30596
Q4	-0.352 (-0.449, -0.255) <0.00001	-0.036 (-0.137, 0.064) 0.47562	-0.078 (-0.577, 0.422) 0.76110	0.114 (-0.434, 0.662) 0.68479
P for trend	<0.00001	0.11763	0.47163	0.94157

Noted:Non-adjusted model adjust for: None

Minimally-adjusted model adjust for: Race/Ethnicity; Poverty income ratio; Age,year; marital status, education level

Fully-adjusted model adjust for: Vitamin D intake (mcg); LDL-cholesterol (mg/dL); Race/Ethnicity; Poverty income ratio; Body mass index,Kg/m2; Alcohol (gm) first day; C-reactive protein (mg/dL); Glycohemoglobin (%); HDL; Hypertension history; Diabetes history; coronary heart disease; stroke; cigarettes per day during past month; Age,year; Marital Status; Average level of physical activity each day; Enlarged prostate; Triglycerides (mg/dL), education level

GAM model:Adjust II model adjust for: Vitamin D intake (mcg) (Smooth); LDL-cholesterol (mg/dL) (Smooth); Race/Ethnicity; Poverty income ratio; Body mass index,Kg/m2(Smooth); Alcohol (gm) first day(Smooth); C-reactive protein (mg/dL) (Smooth); Glycohemoglobin (%)(Smooth); HDL(Smooth); Hypertension history; Diabetes history; coronary heart disease; stroke; cigarettes per day during past month(Smooth); Age,year(Smooth); Marital Status; Average level of physical activity each day; Enlarged prostate; Triglycerides (mg/dL)(Smooth), education level

In order to make the results reliable, we did the following sensitivity analysis: enter X as a categorical variable to ensure the robustness of the results. Since the linear regression equation requires that all the independent variables and the dependent variable must have a linear relationship, when the relationship between the covariate and Y is nonlinear, the result may greatly deviate. Therefore, for the purpose of sensitivity analysis, we adjust all continuous variables in the covariate to the GAM model by the curve. However, although the magnitude and confidence interval of the effect values vary slightly, the direction is consistent with the fully-adjusted model. In addition, these transformations (fitting continuous variables to smoothing) was employed, the results still did not change significantly in the GAM model.

Based on the purpose of sensitivity analysis, dietary protein intake was stratified into a categorical variable by quartile and estimated P for trend (Table 2). In the fully adjusted model, compared with the reference Q1 group, the estimated increase of dietary protein intake in the Q2, Q3, and Q4 group were 0.238 (log2 transformation), − 0.552 and − 0.078, respectively. The P for trend was 0.47163. The results were consistent with the results of dietary protein intake as a continuous variable. Non-equidistant changes in the magnitude of this effect size (B) suggest a possible non-linear relationship between dietary protein intake and PSA.

### Identification of non-linear relationship

**Fig. 2:**
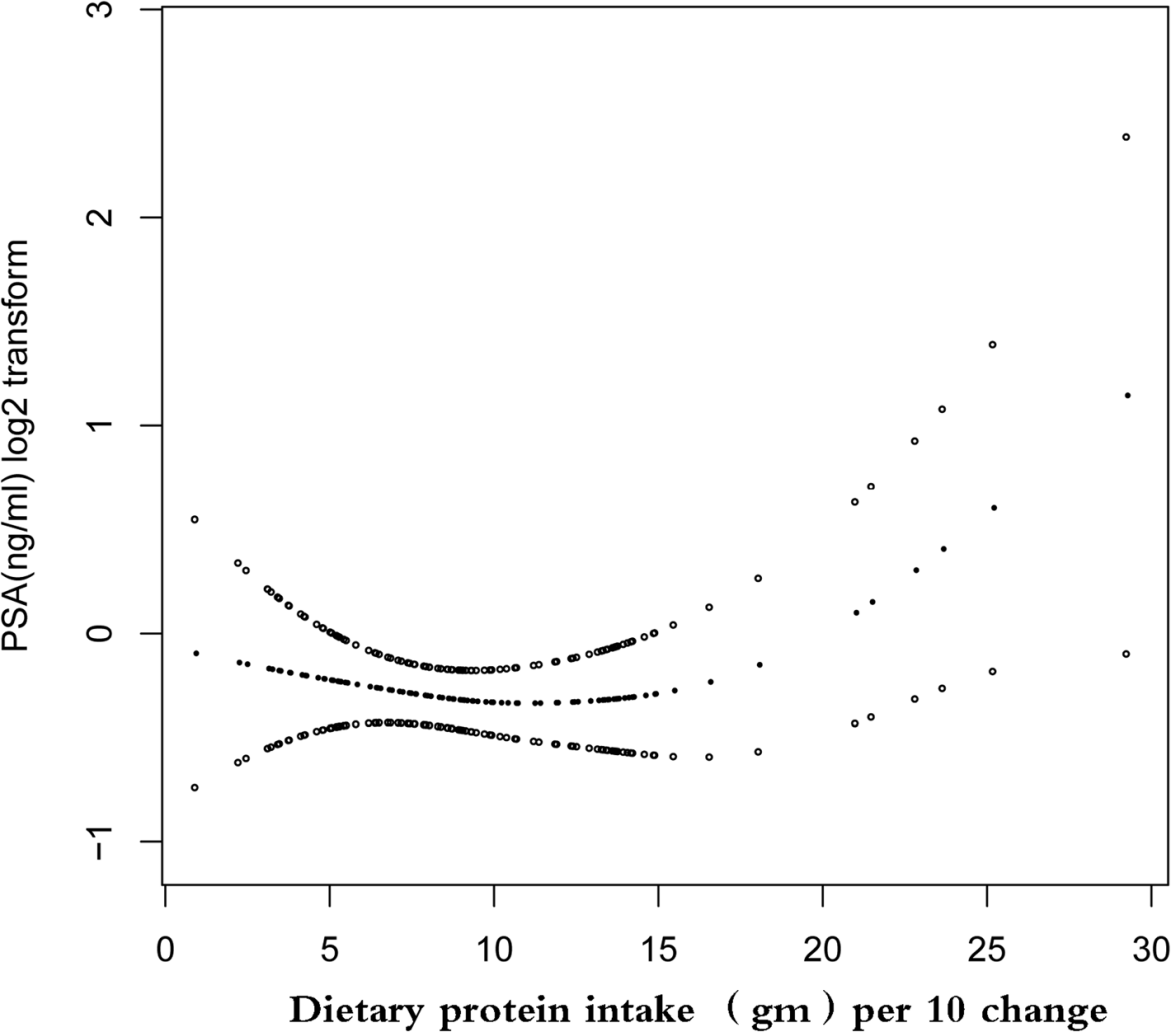
The relationship between dietary protein intake and prostate-specific antigen. A nonlinear relationship between them was detected after adjusting for Vitamin D intake (mcg) (Smooth); LDL-cholesterol (mg/dL) (Smooth); Race/Ethnicity; Poverty income ratio; Body mass index,Kg/m2 (Smooth); Alcohol (gm) first day(Smooth); C-reactive protein (mg/dL) (Smooth); Glycohemoglobin (%)(Smooth); HDL(Smooth); Hypertension history; Diabetes history; coronary heart disease; stroke; cigarettes per day during past month(Smooth); Age, year(Smooth); Marital Status; Average level of physical activity each day; Enlarged prostate; Triglycerides (mg/dL)(Smooth), education level

In the study, the non-linear relationship between dietary protein intake and PSA was also explored (Fig. 2). Using the generalized additive model, the non-linear association between dietary protein intake and PSA was detected. The linear regression model and a two-piecewise linear regression model were compared, and the P for the log-likelihood ratio test is 0.002. This result demonstrates that the two-piecewise linear regression model should be used to fit the model.

By two-piecewise linear regression model and recursive algorithm, we calculated the inflection point was 18.18 g (per 10 changes) (Table 3). On the left of inflection point, the effect size, 95%CI and *P* value were − 0.03(log2 transformation) (− 0.09, 0.02) and 0.2721, respectively. On the right side of the inflection point, a positive association between dietary protein intake and PSA was observed, and the effect size, 95%CI and P value were 0.22(log2 transformation) (0.07, 0.36), *P* = 0.0040). There was somewhat U-shape between dietary protein intake and PSA with dietary protein intake threshold level of 181.8 g. These findings indicated that the threshold effect existed between dietary protein intake and PSA levels.

**Table 3:** Nonlinearity addressing by weighted two-piecewise linear model

Outcome	PSA(ng/ml) log2 transform β (95% CI)
Fitting by weighted linear regression model	0.02 (-0.02, 0.06) 0.2837
Fitting by weighted two-piecewise linear regression model	
Inflection point	18.18
< 18.18	-0.03 (-0.09, 0.02) 0.2721
≥18.18	0.22 (0.07, 0.36) 0.0040
Log likelihood ratio test	0.002

Noted:Independent variable is dietary protein intake per 10 change and dependent variable is PSA(ng/ml log2 transform

Covariates involved in this model was the same as GAM model presented in Table 2

## Discussion

Prostate cancer (PCa) is the most common malignant tumor and the leading cause of cancer deaths for men in Western countries. Therefore, early screening of PCa is helpful for early detection, and early treatment reduces mortality. The current screening of PCa population is mainly based on PSA, so the clarification of the factors affecting PSA will help to improve the quality of screening. Previous literature reports that dietary protein intake is associated with PCa. In addition, there are also reports in the literature that dietary protein intake can affect PSA [7]. Since previous literature has confirmed that dietary protein intake is associated with the development and progression of PCa [7, 8, 20], we speculate that dietary protein intake also affects the level of PSA. In order to verify our hypothesis, the USA NHANES database was used. The database is a large sample of databases that includes a variety of clinical and dietary, sociodemographic, laboratory, and questionnaire data. Therefore, the current work was to explore the relationship between dietary protein intake and PSA among American adults without prostate tumor history. As is shown in the fully adjusted weighted linear regression model (Table 2), dietary protein intake was not related to PSA. However, the non-linear relationship between dietary protein intake and PSA was observed, and the findings indicated that the correlation between dietary protein intake and PSA had a segmental trend. The different relationships of dietary protein intake on PSA were found on the left and right sides of inflection point (dietary protein intake per 10 change > 18.18 g). On the right side of the inflection point, dietary protein intake was positively associated with PSA, but the correlation on the left sides of the inflection point was not statistically significant.

Many studies have reported an association between dietary protein intake and PCa [7, 8, 20, 21]. The recent meta-analysis conducted by Mao Y et al. pooled 12 studies and the combined results revealed that protein intake may be not associated with prostate cancer [20], but in the study, the authors fail to evaluate the nonlinearity association. Fontana L et al. found that dietary protein restriction diet could significantly reduce BMI, increase insulin sensitivity and FGF21 concentration and produce a trend toward reduced PSA levels in human xenograft prostate models [7]. In a randomized trial, Eitan E et al. also reported that dietary protein restriction modifies insulin signaling in circulating extracellular vesicles (EV), which indirectly reflect PSA levels [8]. A study of the association between dietary protein and risk of prostate cancer in the NCI Breast and Prostate Cancer Cohort Consortium (BPC3) a high intake of dairy protein may increase prostate cancer risk by increasing the production of insulin-like growth factor 1 (IGF-1, 21]. However, there was no strong evidence for the multiple interactions of a gene-dietary protein associated with PCa risk. Given that there is a lack of evidence between dietary protein intake and PSA. Therefore, we conducted a secondary study to confirm the hypothesis that higher dietary protein intake is associated with elevated PSA. In the work, we observed a non-linear relationship between dietary protein intake and PSA levels. When dietary total saturated fatty acids were greater than 65.12 g, the dietary protein intake was positively correlated with PSA levels.

Protein is composed of macromolecules made of amino acids and has basic functions in all known biologic processes. Data from epidemiological and human experimental studies suggest that dietary protein restriction is more powerful than calorie or fat restriction in lowering the circulating levels of IGF-1, which could inhibit the PI3K/AKT/mTOR pathway [14, 15]. In addition, IGF/PI3K/Akt/mTOR pathway play a key role in the pathogenesis of PCa [22, 23]. It is possible that the underlying mechanism delineating the association between dietary protein intake and PSA concentration is through IGF-1, which induced changed IGF-1 levels and inhibit the PI3K/AKT/mTOR pathway. Another potential mechanism, which has been supported by recent research, is that dietary protein decreases insulin sensitivity and promote prostate cancer cell tumor growth in animal models, which in turn affect the PSA levels [8, 24]. The potential biological mechanisms could explain the link between dietary protein intake and PSA levels.

The present study exhibited several strengths. Firstly, the research highlight of this study is its large sample size. The study included a large number of 6403 participants, which provides a high statistical power to quantitatively assess the association between dietary protein intake and PSA levels. Second, we have clearly clarified the missing data and performed multiple imputations. Our results demonstrate that there is no significant difference between the data before and after the interpolation, which improves statistical performance and minimizes the bias caused by missing records. Thirdly, we conducted linear and nonlinear regression model to increase comparability, and the results revealed the possibility of a non-linear relationship was detected. Fourthly, GAM was used to elucidate the non-linear relationship. Fifthly, we employed a strict statistical adjustment to minimize residual confounding, which could potentially influence the PSA. Finally, we calculated the inflection point by the recursive algorithm and discovered the saturation effect of dietary protein intake and PSA by two-piecewise linear regression, which provided protein recommendation for dietary guidelines.

The current work presents several limitations that must be considered in interpreting the results. Firstly, the study design was cross-sectional. Due to its inherent limitations, we are unable to derive a causal link between dietary protein intake and PSA was elucidated, and it is difficult to distinguish causality. Secondly, the research population is limited to the American, so the generalizability is geographically restricted. Thirdly, this study is based on a secondary analysis of published data so variables that are not included in the data set cannot be adjusted, such as dihydrotestosterone concentrations.

## Conclusion

The association between dietary protein intake and PSA is non-linear. Dietary protein intake is positively correlated with PSA when PSA is larger than 181.8 g. Large prospective clinical trials with robust methodology are required to confirm our findings.

## Supplementary information

**Additional file 1: Supplement table 1**. The description of missing data. **Supplemental table 2**: Sensitivity comparative analysis between pre-imputation and post-imputation.

## Data Availability

Data can be downloaded from the ‘NHANES’ database (https://www.cdc.gov/nchs/nhanes/index.htm).

## Abbreviations

NHANES: The National Health and Nutrition Examination Survey
PSA: Prostate-specific antigen
PCa: Prostate cancer
PR: Protein Restriction
GAM: Generalized Additive Model
