# Supplementary material for: Is there a non-linear relationship between dietary protein intake and prostate-specific antigen: proof from the national health and nutrition examination survey (2003–2010)
Source: Lipids Health Dis. 2020 May 2;19:82. doi: 10.1186/s12944-020-01234-6 (PMC7195731; doi:10.1186/s12944-020-01234-6)
Supplement: Supplementary file 1 — Additional file 1: Supplement table 1. The description of missing data. Supplemental table 2: Sensitivity comparative analysis between pre-imputation and post-imputation. [file 12944_2020_1234_MOESM1_ESM.docx]

Supplement table 1. The description of missing data.

| **Variables** | **Non-missing** | **Missing** |
| --- | --- | --- |
| VITD | 6437 | 185 |
| LBDLDL | 3053 | 3569 |
| Race/Ethnicity | 6622 | 0 |
| Poverty income ratio | 6187 | 435 |
| Body mass index,Kg/m2 | 6509 | 113 |
| Alcohol (gm) first day | 6344 | 278 |
| HDL | 6622 | 0 |
| C-reactive protein(mg/dL) | 6621 | 1 |
| Hypertension history | 3939 | 2683 |
| Diabetes history | 3838 | 2784 |
| coronary heart disease | 3864 | 2758 |
| stroke | 3864 | 2758 |
| Triglycerides (mg/dL) | 6603 | 19 |
| Enlarged prostate | 4788 | 1834 |
| Age,year | 6622 | 0 |
| Lead (umol/L) | 6617 | 5 |
| Cadmium (nmol/L) | 6617 | 5 |
| Mercury, total (umol/L) | 6617 | 5 |
| Smoked at least 100 cigarettes in life | 3854 | 2768 |

Supplemental table 2: Sensitivity comparative analysis between pre-imputation and post-imputation

| **MI.ITER** | **0** | **1** | **2** | **3** | **4** | **5** | **P-value** | **P-value*** |
| --- | --- | --- | --- | --- | --- | --- | --- | --- |
| Vitamin D | 60.21 (21.72) | 60.23 (21.79) | 60.27 (21.69) | 60.28 (21.66) | 60.29 (21.70) | 60.28 (21.71) | 1.000 | 1.000 |
| LDL-cholesterol | 118.60 (35.22) | 119.78 (35.67) | 119.46 (35.67) | 118.81 (35.57) | 119.16 (35.90) | 119.32 (35.59) | 0.581 | 0.270 |
| Poverty income ratio | 2.74 (1.61) | 2.72 (1.61) | 2.73 (1.62) | 2.73 (1.63) | 2.72 (1.63) | 2.73 (1.62) | 0.976 | 0.995 |
| Body mass index,Kg/m2 | 28.75 (5.55) | 28.77 (5.55) | 28.76 (5.55) | 28.76 (5.55) | 28.75 (5.56) | 28.76 (5.55) | 1.000 | 1.000 |
| Alcohol (gm) first day | 14.40 (34.34) | 14.31 (34.45) | 14.23 (34.37) | 14.48 (34.49) | 14.42 (34.28) | 14.43 (34.27) | 0.999 | 0.968 |
| C-reactive protein(mg/dL) | 0.19 ( 0.01-18.50) | 0.19 (0.01-18.50) | 0.19 ( 0.01-18.50) | 0.19 (0.01-18.50) | 0.19 (0.01-18.50) | 0.19 (0.01-18.50) | 1.000 | 1.000 |
| Glycohemoglobin (%) | 5.89 (1.17) | 5.89 (1.17) | 5.89 (1.17) | 5.89 (1.17) | 5.89 (1.17) | 5.89 (1.17) | 1.000 | 1.000 |
| HDL | 48.58 (14.37) | 48.58 (14.37) | 48.58 (14.37) | 48.58 (14.37) | 48.58 (14.37) | 48.58 (14.37) | 1.000 | 1.000 |
| Age,year | 59.47 (12.79) | 59.47 (12.79) | 59.47 (12.79) | 59.47 (12.79) | 59.47 (12.79) | 59.47 (12.79) | 1.000 | 1.000 |
| Triglycerides (mg/dL) | 133.00 ( 21.00-2693.00) | 133.00 (-295.03-2693.00) | 133.00 ( -75.64-2693.00) | 133.00 (-175.92-2693.00) | 133.00 (-208.16-2693.00) | 133.00 ( -93.90-2693.00) | 1.000 | 1.000 |
| Race/Ethnicity |  |  |  |  |  |  | 1.000 | - |
| Mexican American | 1239 (18.71%) | 1239 (18.71%) | 1239 (18.71%) | 1239 (18.71%) | 1239 (18.71%) | 1239 (18.71%) |  |  |
| Other Hispanic | 387 ( 5.84%) | 387 ( 5.84%) | 387 ( 5.84%) | 387 ( 5.84%) | 387 ( 5.84%) | 387 ( 5.84%) |  |  |
| Non-Hispanic White | 3504 (52.91%) | 3504 (52.91%) | 3504 (52.91%) | 3504 (52.91%) | 3504 (52.91%) | 3504 (52.91%) |  |  |
| Non-Hispanic Black | 1241 (18.74%) | 1241 (18.74%) | 1241 (18.74%) | 1241 (18.74%) | 1241 (18.74%) | 1241 (18.74%) |  |  |
| Other Race - Including Multi-Racial | 251 ( 3.79%) | 251 ( 3.79%) | 251 ( 3.79%) | 251 ( 3.79%) | 251 ( 3.79%) | 251 ( 3.79%) |  |  |
| Hypertension history |  |  |  |  |  |  | 0.221 | - |
| Yes | 1671 (42.42%) | 3076 (46.45%) | 3080 (46.51%) | 3064 (46.27%) | 3198 (48.29%) | 3202 (48.35%) |  |  |
| No | 2268 (57.58%) | 3546 (53.55%) | 3542 (53.49%) | 3558 (53.73%) | 3424 (51.71%) | 3420 (51.65%) |  |  |
| Diabetes history |  |  |  |  |  |  | 0.191 | - |
| Yes | 626 (16.31%) | 1351 (20.40%) | 1298 (19.60%) | 1319 (19.92%) | 1318 (19.90%) | 1387 (20.95%) |  |  |
| No | 3212 (83.69%) | 5271 (79.60%) | 5324 (80.40%) | 5303 (80.08%) | 5304 (80.10%) | 5235 (79.05%) |  |  |
| coronary heart disease |  |  |  |  |  |  | 0.063 | - |
| Yes | 283 ( 7.32%) | 833 (12.58%) | 795 (12.01%) | 795 (12.01%) | 803 (12.13%) | 770 (11.63%) |  |  |
| No | 3581 (92.68%) | 5789 (87.42%) | 5827 (87.99%) | 5827 (87.99%) | 5819 (87.87%) | 5852 (88.37%) |  |  |
| stroke |  |  |  |  |  |  | 0.082 | - |
| Yes | 180 ( 4.66%) | 425 ( 6.42%) | 482 ( 7.28%) | 482 ( 7.28%) | 469 ( 7.08%) | 515 ( 7.78%) |  |  |
| No | 3684 (95.34%) | 6197 (93.58%) | 6140 (92.72%) | 6140 (92.72%) | 6153 (92.92%) | 6107 (92.22%) |  |  |
| **Smoked at least 100 cigarettes in life** |  |  |  |  |  |  | 0.609 | - |
| Yes | 2431 (63.08%) | 4108 (62.04%) | 4104 (61.98%) | 4055 (61.24%) | 4092 (61.79%) | 4100 (61.91%) |  |  |
| No | 1423 (36.92%) | 2514 (37.96%) | 2518 (38.02%) | 2567 (38.76%) | 2530 (38.21%) | 2522 (38.09%) |  |  |
| **Physical Activity**  **(MET-based rank) (%)** |  |  |  |  |  |  | 0.077 | - |
| Sits | 991 (26.19%) | 1508 (22.77%) | 1548 (23.38%) | 1607 (24.27%) | 1588 (23.98%) | 1629 (24.60%) |  |  |
| Walks | 1867 (49.34%) | 3227 (48.73%) | 3183 (48.07%) | 3195 (48.25%) | 3243 (48.97%) | 3239 (48.91%) |  |  |
| Light loads | 617 (16.31%) | 1253 (18.92%) | 1263 (19.07%) | 1220 (18.42%) | 1189 (17.96%) | 1161 (17.53%) |  |  |
| Heavy work | 309 ( 8.17%) | 634 ( 9.57%) | 628 ( 9.48%) | 600 ( 9.06%) | 602 ( 9.09%) | 593 ( 8.95%) |  |  |
| **Enlarged prostate** |  |  |  |  |  |  | 0.809 | - |
| Yes | 836 (17.55%) | 1180 (17.82%) | 1142 (17.25%) | 1125 (16.99%) | 1127 (17.02%) | 1145 (17.29%) |  |  |
| No | 3928 (82.45%) | 5442 (82.18%) | 5480 (82.75%) | 5497 (83.01%) | 5495 (82.98%) | 5477 (82.71%) |  |  |
| **Education (%)** |  |  |  |  |  |  | 1.000 | - |
| less than high  school | 1155 (17.47%) | 1157 (17.47%) | 1158 (17.49%) | 1159 (17.50%) | 1160 (17.52%) | 1157 (17.47%) |  |  |
| high school | 2488 (37.63%) | 2493 (37.65%) | 2493 (37.65%) | 2491 (37.62%) | 2490 (37.60%) | 2494 (37.66%) |  |  |
| more than high  school | 2969 (44.90%) | 2972 (44.88%) | 2971 (44.87%) | 2972 (44.88%) | 2972 (44.88%) | 2971 (44.87%) |  |  |
| **Martial Status (%)** |  |  |  |  |  |  | 1.000 | - |
| married | 4493 (67.94%) | 4500 (67.96%) | 4500 (67.96%) | 4498 (67.93%) | 4500 (67.96%) | 4500 (67.96%) |  |  |
| single | 1788 (27.04%) | 1790 (27.03%) | 1790 (27.03%) | 1791 (27.05%) | 1790 (27.03%) | 1790 (27.03%) |  |  |
| living with partner | 332 ( 5.02%) | 332 ( 5.01%) | 332 ( 5.01%) | 333 ( 5.03%) | 332 ( 5.01%) | 332 ( 5.01%) |  |  |
